# Perceptions of Clinical Dental Students Toward Online Education During the COVID-19 Crisis: An Egyptian Multicenter Cross-Sectional Survey

**DOI:** 10.3389/fpsyg.2021.704179

**Published:** 2022-01-07

**Authors:** Reham Hassan, Ayman R. Khalifa, Tarek Elsewify, Mohamed G. Hassan

**Affiliations:** ^1^Department of Endodontics, Faculty of Dentistry, Egyptian Russian University, Cairo, Egypt; ^2^Department of Endodontics, Faculty of Dentistry, Minia University, El-Minia, Egypt; ^3^Department of Orthodontics, Faculty of Dentistry, October 6 University, Giza, Egypt; ^4^Department of Endodontics, Faculty of Dentistry, Ain Shams University, Cairo, Egypt; ^5^Department of Restorative Dental Sciences, College of Dentistry, Gulf Medical University, Ajman, United Arab Emirates; ^6^Department of Orthodontics, Faculty of Dentistry, Assiut University, Assiut, Egypt; ^7^Division of Bone and Mineral Diseases, Department of Medicine, School of Medicine, Washington University in St. Louis, St. Louis, MO, United States

**Keywords:** COVID-19, dental education, clinical education, dental students, online education, coronavirus

## Abstract

**Objectives:** To evaluate the perceptions of clinical dental students on the role of online education in providing dental education during the COVID-19 crisis.

**Materials and Methods:** A cross-sectional online survey was sent to four Egyptian dental schools from the 20th of January 2021 to the 3rd of February 2021. Survey questions included the demographics, uses, experiences, perceived benefits, and barriers of distance learning in dentistry during the COVID-19 pandemic. Responses were collected from the clinical dental school students. Categorical data were presented as frequencies (*n*) and percentages (%) and were analyzed using Fisher’s exact test.

**Results:** Three hundred thirty-seven clinical dental students across four Egyptian dental schools responded. Most students used either Google Classroom or Microsoft Teams to access the online content. The data showed that the COVID-19 pandemic affected the academic performance of most participants (97.4%) with varying degrees. On average, students were neutral when asked to rate the online lectures, but did not find online practical education as effective (81.3%) as online theoretical teaching. The commonly described barriers to online teaching included loss of interaction with educators, inappropriateness in gaining clinical skills, and the instability of the internet connection.

**Conclusion:** Despite the reported benefits, clinical dental students in Egypt preferred the hybrid approach in dental education as distance learning represented a prime challenge to gain adequate clinical dental skills.

## Introduction

In December 2019, COVID-19 was first identified in Wuhan, China (Li et al., 2020). The COVID-19 outbreak rapidly spread worldwide and on the 15th of March 2020, the WHO announced COVID-19 as a pandemic (WHO/Europe, 2020). The overall number of confirmed cases and mortalities are 141,754,944 and 3,025,835, respectively, in 216 countries as of the 20th of April 2021 (WHO Coronavirus Disease (COVID-19) Dashboard, 2020).

COVID-19 has affected the educational process at all levels. The majority of the educational institutions during the first and the second waves closed campuses and transitioned from physical attendance to virtual learning (Hassan and Amer, 2021). Early in this pandemic, many countries, including Egypt, began to implement precautionary measures, such as social distancing and lockdowns of educational institutions in order to control and mitigate the pandemic (Social Distancing, 2020). Medical institutions quickly adapted to the COVID-19 pandemic. Dental and oral health education services became greatly affected due to the dental team’s proximity to the patient and aerosols during routine dental therapeutic procedures (Shehata et al., 2020). Dental institutions recognized the challenges this pandemic presented to dental students (Barabari and Moharamzadeh, 2020; Hung et al., 2021). Specifically, the pandemic affected clinical training for dental students, which could delay them from taking regional dental board exams or completing other graduation requirements (Hassan and Amer, 2021; Hung et al., 2021). In Egypt, the Ministry of Higher Education and Scientific Research authorized the replacement of ongoing classroom teaching with e-learning for both undergraduate and postgraduate education using various software and interactive online platforms (Hassan and Amer, 2021). Dental institutions in Egypt encountered many challenges making this transition to online education. One of these challenges includes accessibility by the low-income population of the developing countries (World Economic Outlook Databases, 2021), which does not always have compatible electronic devices nor stable access to an Internet connection (Egypt: number of internet users, 2019; Online Education, 2021). Furthermore, the resumption of in-person practical dental courses (laboratory, pre-clinical, and clinical), which are important for the development of skills of dentist training, are fundamentally important to assure educational and therapeutic services in a safe environment for students, faculty, and patients.

As dental schools adapt to the sudden transition in dental education, little is known about how this shift has affected students, and whether e-learning can be considered as effective as the usual face-to-face tutorials and should continue to exist post-pandemic. The media and the UNESCO have raised concerns regarding the impact of these changes to university teaching on student education in the COVID-19 and post-COVID-19 world (Lewin, 2020). During the year 2020, mounting research has examined the application of e-learning styles in medical and dental academic institutions in response to the COVID-19 crisis (Al-Balas et al., 2020; Bennardo et al., 2020; Brar et al., 2021; Herr et al., 2021). In Italy, dental schools moved classes and examinations into virtual platforms (Bennardo et al., 2020). This shift was followed by delaying the beginning of the new semester to allow professors and educators time to prepare digital content according to the curriculum changes. The number of online courses offered from 21 Chinese academic dental institutions moved from 33 online courses before the pandemic into 119 online courses within 3 weeks (Liu et al., 2020). Other studies have focused on students’ feedback about online dental classes (Van Doren et al., 2020; Cheng et al., 2021). However, we believe that the field still needs to understand the different variables that could affect the students’ feedback toward online classes. Moreover, dental educators should be willing to receive students’ perspectives toward this massive change in dental education and how they can handle these serious challenges. Considering the “students’ perspective” and integrating their views is fundamental for administrators, faculty, and other policymakers as they re-envision dental education in a new “virtual reality.”

The purpose of this study was to determine the effects of the COVID-19 pandemic on the perceptions of Egyptian clinical dental students. We conducted an online survey on senior dental students in both public and private Egyptian dental academic institutions. The survey addressed the online education strategies and suggestions for better dental educational approaches. Challenges related to clinical dental education were also analyzed. We believe that dental schools need to consider sharing and documenting their experiences, and the challenges they faced during this pandemic in both published research. Internationally, this combined evidence will help dental educators to improve educational systems in both pre-clinical and clinical dental education.

## Materials and Methods

### Setting and Participants

The project was conducted with the participation of senior dental students in four universities [two public (South Valley University, Ain Shams University) and two private (Egyptian Russian University, October 6 University) universities]. All the participating universities are accredited by the Egyptian Supreme Council of Universities and apply the same educational modalities. Students were asked to answer the survey questionnaire for research purposes. A power analysis was performed to ensure adequate power for our statistical testing on the prevalence of the perceptions of clinical dental students on the role of online education in providing dental education during the COVID-19 pandemic. Power analysis parameters were obtained from the results of Varvara et al. (2021). Using a confidence interval of 95% and a margin of error of 5% with finite population correction, the predicted sample size (*n*) was a total of 235 responses. Sample size calculation was performed using Epi info for Windows version 7.2 (Dean et al., 2011).

### Survey Questionnaire

This descriptive cross-sectional study was conducted online at the national level. A 20 item survey was devised following a literature search on current online education methods and the effects of COVID-19 on dental and medical education (Dost et al., 2020; Mahdy, 2020). The questionnaire was drafted and informally discussed with an interdisciplinary team^1^ to confirm that the survey questions were aligned with study aims. The questions explored the following three sections: the demographic characteristics of participants, the perceived effect of the COVID-19 pandemic on the academic performance of the students and the online education experience during the lockdown, and the perceived effect of the pandemic on the student’s clinical performance. The questionnaire was created using Google Forms, and an initial trial was given to 30 participants to ensure that the draft questionnaire was understandable. The questionnaire was then distributed by the authors *via* each school’s online platform and social media feeds. The questionnaire was accessible *via* an anonymous link and open for a two-week period.^2^

### Participant Consent and Ethical Considerations

The current study was approved by the Research Ethics Committee of Ain Shams University, Cairo, Egypt (Approval # 08092020). Participants were informed that participation was voluntary and clarification was presented that all data would be non-identifiable and would only be used for research purposes.

### Data Analysis

The survey responses were exported from Google Form to Microsoft Excel (Office 365). Descriptive statistics were calculated for the survey responses to explore patterns in responses. Categorical data were presented as frequencies (*n*) and percentages (%) and were analyzed using Fisher’s exact test. Quantitative data were presented as mean and standard deviation values. Values of *p* were adjusted for multiple comparisons utilizing Bonferroni correction. The significance level was set at *p* ≤ 0.05 for all tests. Statistical analysis was performed with R statistical analysis software version 4.0.3 for Windows (RStudio Team, 2015).

## Results

### Cohort Demographics

**Figure 1:**
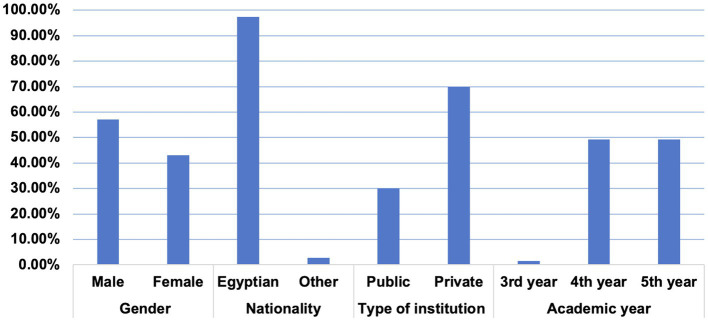
Bar chart showing percentage values for demographic characteristics.

A total of 345 responses were retrieved. Eight responses were excluded due to missing or invalid data. The remaining 337 responses were from four different public and private Egyptian dental institutions (two each; Table 1; Figure 1). Of the 337 responses collected, there were more male participants (57%, *n* = 192) than female participants (43%, *n* = 145). There were more private dental school students (70%, *n* = 236) than public dental school students (30%, *n* = 101). About 50% (*n* = 166, 49.3%) of the participants were 5th year students while 49.3% (*n* = 166) were 4th year dental students (Table 1; Figure 1).

**Table 1:** Frequency and percentage (%) for answers to demographic characteristics.

Question	Answers	**n**	%	Value of **p**
Gender	Male	192	57.0%	0.011^*^
	Female	145	43.0%	
Nationality	Egyptian	328	97.3%	<0.001^*^
	Other	9	2.7%	
Type of institution	Public	101	30.0%	<0.001^*^
	Private	236	70.0%	
Academic year	3rd year	5	1.5%	<0.001^*^
	4th year	166	49.3%	
	5th year	166	49.3%	

* Significant (*p* ≤ 0.05).

ns, non-significant (*p* > 0.05).

### The Effect of the COVID-19 Pandemic on the Academic Dental Performance

**Figure 2:**
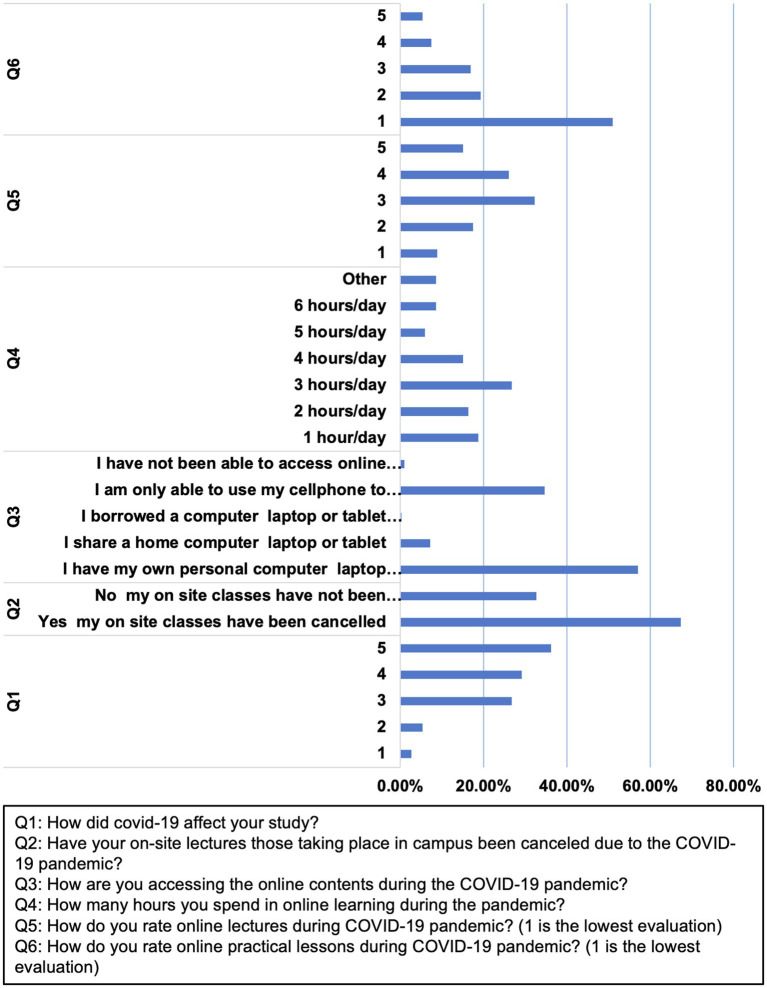
Students’ responses to the survey on the impact of COVID-19 pandemic on the academic performance. The corresponding questions are as follows: Q1: How did COVID-19 affect your study? Q2: Were your on-site lectures on campus canceled due to the COVID-19 pandemic? Q3: How are you accessing the online content during the COVID-19 pandemic? Q4: How many hours do you spend in online education during the pandemic? Q5: How do you rate online lectures during COVID-19 pandemic? (1 is the lowest evaluation) Q6: How do you rate online practical lessons during COVID-19 pandemic? (1 is the lowest evaluation).

The majority of the participants (65.3%) believed that COVID-19 pandemic affected their academic performance. Given the current COVID-19 practices, 67.4% of the participants had their on-site classes canceled. Most students expressed overall satisfaction (73.5%) with online lectures. The online practical sessions had an opposite trend with 70.3% overall dissatisfaction. Most participants used personal laptops or tablet computers (57%) to participate in online classes, spending 3 h on average (61.7%) every day (Table 2; Figure 2).

**Table 2:** Frequency and percentage (%) for answers for assessing the impact of COVID-19 pandemic on the academic performance.

S. No.	Question	Answers	**n**	%	Value of **p**
1.	How did covid 19 pandemic affect your study?	1	9C	2.7%	<0.001^*^
		2	18C	5.3%	
		3	90B	26.7%	
		4	98AB	29.1%	
		5	122A	36.2%	
2.	Were your on-site lectures on campus canceled due to the COVID-19 pandemic?	Yes, my on-site classes have been canceled	227A	67.4%	<0.001^*^
		No, my on-site classes have not been canceled	110B	32.6%	
3.	How are you accessing online course content during the covid 19 pandemic?	I have my own personal computer, laptop or tablet	192A	57.0%	<0.001^*^
		I share a home computer, laptop or tablet	24C	7.1%	
		I borrowed a computer, laptop or tablet from someone outside of my home	1D	0.3%	
		I am only able to use my cellphone to access content	117B	34.7%	
		I have not been able to access online course content	3D	0.9%	
4.	How many hours do you spend in online learning during the pandemic?	1 h/day	63AB	18.7%	<0.001^*^
		2 h/day	55B	16.3%	
		3 h/day	90A	26.7%	
		4 h/day	51B	15.1%	
		5 h/day	20C	5.9%	
		6 h/day	29C	8.6%	
		Other	29C	8.6%	
5.	How do you rate online lectures during covid 19 pandemic? 1 is the lowest evaluation	1	30C	8.9%	<0.001^*^
		2	59B	17.5%	
		3	109A	32.3%	
		4	88A	26.1%	
		5	51B	15.1%	
6.	How do you rate online practical lessons during covid 19 pandemic? 1 is the lowest evaluation	1	172A	51.0%	<0.001^*^
		2	65B	19.3%	
		3	57B	16.9%	
		4	25C	7.4%	
		5	18C	5.3%	
7.	Which virtual learning tools did you use during the covid 19 pandemic?	University Platform	106B	31.5%	<0.001^*^
		Online Classes	170A	50.4%	
		Educational Websites	5D	1.5%	
		Youtube videos	25C	7.4%	
		E-books	12CD	3.6%	
		Educational applications	14CD	4.2%	
		Other	5D	1.5%	
8.	Which online learning tool do you use during the covid 19 pandemic?	Zoom	9CD	2.7%	<0.001^*^
		Microsoft Teams	63B	18.7%	
		moodle	12C	3.6%	
		Google classroom	251A	74.5%	
		Social Media Networks (ex: Facebook)	2D	0.6%	
9.	Which of these forms of online lectures has been the most dominant please select only one?	Online in real time video conference	56B	16.6%	<0.001^*^
		Online with a video recording not in real time	208A	61.7%	
		Online with an audio recording not in real time	62B	18.4%	
		Online by sending presentations to students with no video or audio recording	11C	3.3%	
10.	What is the evaluation method used during the covid 19 pandemic?	Online exams remotely	243A	72.1%	<0.001^*^
		Online exams at the college’s testing center	14C	4.2%	
		Assignments only	36B	10.7%	
		Written exam at college	44B	13.1%	
11.	Which method of evaluation do you find most accurate and convenient?	Assignments only	15B	4.5%	<0.001^*^
		Online exams at the college’s testing center	25B	7.4%	
		Online exams remotely	163A	48.4%	
		Written exam at college	134A	39.8%	
12.	Would you prefer to get back to onsite lectures or continue to use online learning after the end of the pandemic?	Get back to onsite lectures	165A	49.0%	<0.001^*^
		Continue to use online learning	172A	51.0%	

* Significant (*p* ≤ 0.05).

Different letters indicate a statistically significant difference within the same question; ns, non-significant (*p* > 0.05).

Nearly 50% of the participants (73.6%) used online classes. Google Classroom was the most used platform for online classes (74.5%), followed by Microsoft Teams (18.7%). The classes were pre-recorded and uploaded on the platform for most of the participants (80.1%; Table 2; Figure 2). Although more than 70% of the participants had their exams remotely online, some of the students (39.8%) expressed their preference of attending the exams in person.

### Assessing the Impact of COVID-19 Pandemic on the Clinical Training of Students

**Figure 3:**
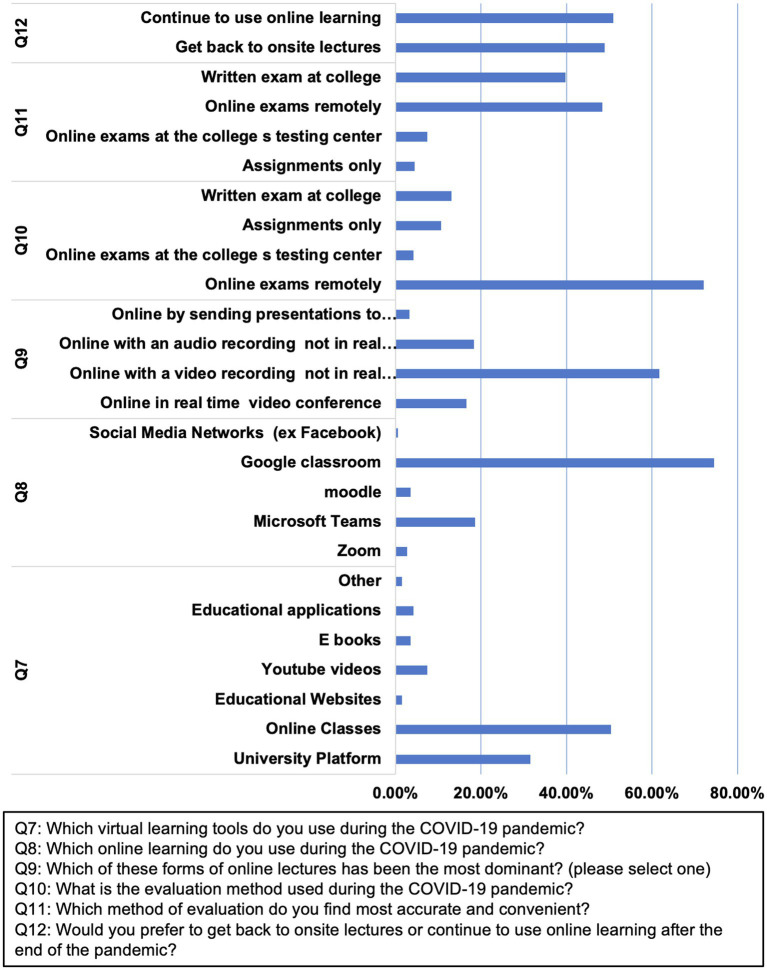
Students’ responses to the survey on the impact of COVID-19 pandemic on the academic performance. The corresponding questions are as follows: Q7: Which virtual learning tools do you use during the COVID-19 pandemic? Q8: Which online learning do you use during the COVID-19 pandemic? Q9: Which of these forms of online lectures has been the most dominant? (Please select one) Q10: What is the evaluation method used during the COVID-19 pandemic? Q11: Which method of evaluation do you find most accurate and convenient? Q12: Would you prefer to get back to on-site lectures or continue to use online education after the end of the pandemic?

**Figure 4:**
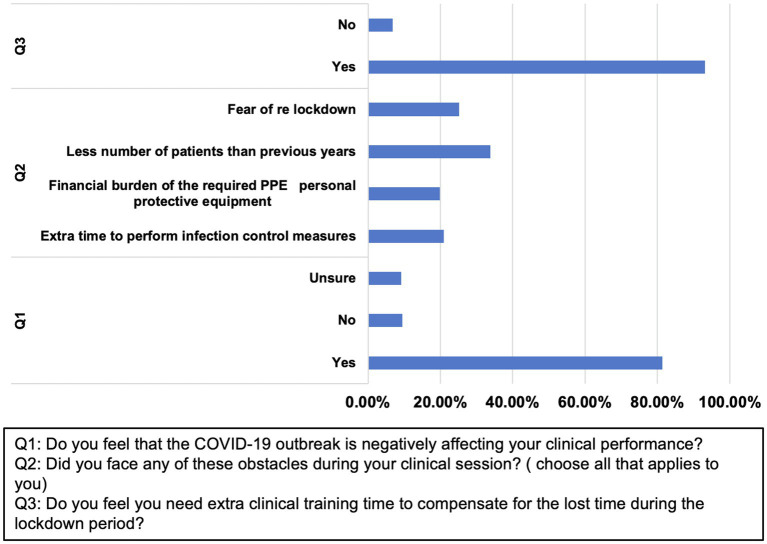
Student’s responses to the survey on the impact of COVID-19 pandemic on the clinical dental performance. The corresponding questions are as follows: Q1: Do you feel that the COVID-19 outbreak is negatively affecting your clinical performance? Q2: Did you face any of these obstacles during your clinical session? (Choose all that applies to you) Q3: Do you feel you need extra clinical training time to compensate for the lost time during the lockdown period?

Most students (81.3%) reported more negative responses related to the effect of the pandemic on their clinical performance (Table 3; Figure 3). Students expressed that the COVID-19 pandemic affected them negatively with a smaller number of patients available for clinical training (33.9%), in addition to the fear of the re-lockdown (25.2%). Most of the dental students (93%) participating in the survey expressed the need to have additional clinical training after graduation to compensate for the time lost during the lockdown (Table 3; Figure 4).

**Table 3:** Frequency and percentage (%) for answers for assessing the impact of COVID-19 pandemic on the Clinical training of students.

S. No.	Question	Answers	**n**	%	Value of **p**
1.	Do you feel that the covid 19 outbreak is negatively affecting your clinical performance?	Yes	274A	81.3%	<0.001^*^
		No	32B	9.5%	
		Unsure	31B	9.2%	
2.	Do you feel that the covid 19 outbreak is negatively affecting your clinical performance?	Extra time to perform infection control measures	158BC	20.9%	<0.001^*^
		Financial burden of the required PPE personal protective equipment	151C	19.9%	
		Less number of patients than previous years	257A	33.9%	
		Fear of re lockdown	191B	25.2%	
3.	Do you feel you need extra clinical training time to compensate for the lost time during the lockdown period?	Yes	314A	93.2%	<0.001^*^
		No	23B	6.8%	

* Significant (*p* ≤ 0.05).

Different letters indicate a statistically significant difference within the same question; ns, non-significant (*p* > 0.05).

### The Participants’ Responses Regarding the Common Problems With Online Education Could Be Summarized as Follow:

- The speed and cost of the Internet in Egypt prevented the delivery of study materials to students, and the Internet connection was insufficient to download and view the lectures.
- The quality of the recorded videos was not great (e.g., presence of noise and/or low resolution).
- The interactive component was missing The absence of discussions made online learning less interactive due to insufficient interactions between students and professors, which made the lectures boring for them, and easy to lose concentration.
- There was a miscommunication between the administration and academic departments, there were problems with the lectures schedule.
- The timing and the organization of the online exams were problematic. Moreover, the online educational platforms did not allow the students to resume the exams when Internet connection was lost.
- It is difficult to teach clinical subjects using online platforms.

### The Students’ Recommendations Regarding Improvements of the Online Education Were Summarized as Follows:

- Provide training for lecturers on e-learning tools and computer skills.
- Improve the voice recording for lectures by using a separate microphone and compress recorded lectures before uploading them.
- Enhance the interaction between students and teachers, provide real-time online meetings to discuss the subject rather than a recorded video.
- Provide 10–15 min at the end of each lecture for students’ questions.
- Provide a well-organized timetable that suits both lecturers and students.
- Weekly online assessment to follow students after each lecture.
- Provide virtual resources to mimic the laboratory work or live streaming directly from the laboratory.
- Provide practical learning through interactive tools, such as videos and 3D animation.
- More time is required to develop clinical skills to compensate for the lost time during the lockdown.

## Discussion

With the escalation of the COVID-19 pandemic, it is foreseeable that many dental academic institutions have shifted to online education platforms instead of in-person education (Hassan and Amer, 2021; Herr et al., 2021). In this survey, we explored the major challenges during the transition into distance learning, overall satisfaction as well as a future vision toward online education in dental education among clinical dental students in public and private Egyptian dental schools.

Our data showed that 337 participants from four Egyptian dental schools answered the survey. Survey responders were 57% male and 43% female. Although these levels are similar to the demographics of the Egyptian population (45.5% women and 55.5% men; Egypt Data Portal, 2021) they are different than the population of dental students in the United States, which comprises 38% women and 62% men (Scarbecz and Ross, 2002). The current data showed that COVID-19 pandemic lockdown affected the academic performance of most participants to varying degrees and only a small percentage of participants reported that the pandemic had no effect on their academic performance. This is in agreement with previous studies, which reported that COVID-19 pandemic has had a profound impact on medical, dental, and veterinary students (Iyer et al., 2020; Mahdy, 2020; Rose, 2020).

Unlike recently published studies, the most used online platform among the participants in our survey was Google Classroom, followed by Microsoft Teams. This finding contrasts with previous work where ZOOM was reported to be the most commonly used online platform (Al-Balas et al., 2020; Ahmed et al., 2021; Brar et al., 2021; Herr et al., 2021; Temash et al., 2021). Herr et al. (2021) found that ZOOM was the most used platform among South Korean dental students (Herr et al., 2021). We hypothesize two reasons for the differences. First, Google Classroom is a freely accessible platform, whereas ZOOM requires a subscription to extend meetings longer than the 40-min time limit. This lower barrier of Google Classroom may have helped the transition to readily available online educational content in the absence of a well-established educational platform in the majority of the Egyptian institutions. Secondly, there was a high level of cooperation between the Egyptian Ministry of Higher Education and Scientific Research and Microsoft Corporation to provide Teams educational package for the public universities which helped contribute to the high level of usage of Microsoft Teams that we observed in our survey (Ministry of Higher Education and Scientific Research and Microsoft Egypt joined forces to empower fresh graduates with digital skills through “Bina’a Insaan” initiative – Middle East and Africa News Center, 2021).

Regarding the accessibility to the online content, most of the students used their own laptops or tablets. It is worth mentioning that most of the students (91.5%) owned an electronic device to access the online content. These results are in agreement with the results from different surveys among German, South Korean, and Brazilian dental students (Schlenz et al., 2020; Herr et al., 2021; Silva et al., 2021). However, when the students were given the opportunity to elaborate their views of the common problems associated with online education, the speed and cost of the Internet in Egypt were one of the main reasons that prevented the proper delivery of study materials to students (Online Education, 2021). Economic distress may be one explanation for the survey results as studies have shown that students in economic distress are more likely to have poor or no Internet access because they cannot afford the cost of a laptop/computer, the Internet connection, or because they live in regions or neighborhoods with low connectivity (Universities tackle the impact of COVID-19 on disadvantaged students, 2021).

Although the integration of online education in Egyptian dental programs is still new, most of the participants were neutral when asked to rate the online lectures. Online education is known to have many advantages such as the accessibility of the educational materials, the flexibility of studying with the possibility of repeating content, and ultimately enabling the students to customize different study plans. In a survey published in 2021, dental students in the United States rated their online curriculum positively, with 87.6% reporting a high degree of comfort adapting to technology and only 12.4% feeling neutral. No students reported being uncomfortable with the online technology, as 72.0% of students had completed at least one online course prior to dental school and 34.8% had reported completing at least four online classes prior to the COVID-19 pandemic (Hung et al., 2021). A systematic review of 59 studies suggested that online education is equivalent to traditional teaching in terms of knowledge gained, skills gained, and student satisfaction (George et al., 2014).

In contrast to the traditional classroom courses, many students considered their clinical performance to be negatively affected by the transition to online platforms due to the COVID-19 pandemic, with the reduced exposure to patients hindering their development of clinical skills and professionalism. Students lost almost 4 months of clinical practice with the cancelation of the final practical hands-on assessment. Survey participants perceived that they missed important learning experiences, particularly the clinical sessions. The effect of the COVID-19 pandemic on the clinical skills of final year students who did not have enough time to compensate for lost educational time is difficult to assess and consequences may only become evident in the subsequent years. Even after schools re-opened, students observed a negative impact on patient attendance at appointments during the beginning of the COVID-19 pandemic. A recent study reported a significant reduction in the use of emergency dental services by 38% at the start of the COVID-19 outbreak in China, suggesting COVID-19 influenced people’s dental care-seeking behavior (Van Doremalen et al., 2020). This might be because people were reluctant to go outside and leave the house, and were less willing to seek dental care. It is important to mention that the prolonged suspension of clinical training is likely to impact the clinical confidence and competence of dental students.

While there is still no substitute for clinical training, which is the core curriculum of dental schools, survey participants suggested that online case-based discussions, treatment planning exercises, and extra clinical sessions at the end of the year may be helpful to make up for the lost learning due to the pandemic disruptions. Virtual clinical learning innovations are currently being proposed to complement standard clinical practice as a safe way to acquire practical clinical skills through simulation exercises, without direct contact with patients to minimize COVID-19 transmission risks. Such systems provided the tutor and students with continuous and integrated feedback (Hollis et al., 2011). Virtual reality (VR) simulators have the capability of tactile feedback, which allows students to touch and feel the dental tissue virtually. Studies have shown that the use of VR has improved the acquisition of manual dexterity in dentistry courses in the operative area (Miyazono et al., 2019; Morales-Vadillo et al., 2019). However, a large investment from colleges is required to offer this type of teaching methodology.

### Future Improvements of Online Education in Egypt

Based on the students’ feedback, dental schools are required to provide faculties not only with intense training on the technical aspects of the virtual platform itself but also on basic principles of instructional design for effective online delivery to promote student engagement and appropriate assessment methodology. Additional infrastructure and resources are required to support the complete transition.

### Limitations and Future Work

As far as we know, this study is the first to investigate the impact of COVID-19 on clinical dental education across Egypt, with responses from four dental schools. One of the strengths of this survey is its diversity of senior dental students across private and public Egyptian dental academics. Furthermore, the recruitment of the clinical dental students for this survey distribution *via* a range of methods minimized potential response bias. However, this study also had some limitations. Some dental schools may have been disproportionately represented with larger numbers of responses from some schools, for example, Egyptian Russian University. For this reason, the results should be interpreted cautiously before being generalized to the dental education community. Finally, answering some questions of this questionnaire depended on students’ memory which may be subjected to reporting and recall bias.

## Conclusion

This cross-sectional survey highlighted the perception of clinical dental students toward online education during the COVID-19 crisis in Egypt. The participants in the current study agreed that the COVID-19 pandemic significantly affected dental education with varying degrees. According to the students’ answers, e-learning can act as a valuable tool that could help clinical dental students to follow-up lectures and journal clubs. Despite the reported benefits, dental students preferred the hybrid approach in dental education as distance learning represented a prime challenge to gain adequate clinical dental skills. Since the majority of the final years' courses are clinical, students highlighted the difficulty to fulfill the requirements for clinical competencies only using online platforms.

## Data Availability Statement

The raw data supporting the conclusions of this article will be made available by the authors, without undue reservation.

## Ethics Statement

The studies involving human participants were reviewed and approved by Research Ethics Committee, Faculty of Dentistry, Ain Shams University. The patients/participants provided their written informed consent to participate in this study.

## Author Contributions

All authors have read and approved the final article. MGH and RH conceived and designed the study. MGH, RH, TE, and AK collected the data and wrote and revised the manuscript. MGH analyzed the data.

## Conflict of Interest

The authors declare that the research was conducted in the absence of any commercial or financial relationships that could be construed as a potential conflict of interest.

## Publisher’s Note

All claims expressed in this article are solely those of the authors and do not necessarily represent those of their affiliated organizations, or those of the publisher, the editors and the reviewers. Any product that may be evaluated in this article, or claim that may be made by its manufacturer, is not guaranteed or endorsed by the publisher.

## Footnotes

^1^ https://docs.google.com/forms/d/e/1FAIpQLScJ291UIjEcFV3UvtEeWgbxZnt_1TRXsgz9FRw8qWMvCeJ8zA/viewform?usp=sf_link

^2^ https://docs.google.com/forms/d/e/1FAIpQLSdOyiEnR543YvHKdm2fMSrxX0oV3lJN_rSumUsSpOd56z-uag/viewform?usp=sf_link
